# Evaluation of seasonal and monthly variation and location of deep vein thrombosis

**DOI:** 10.1590/1677-5449.202300802

**Published:** 2023-10-13

**Authors:** Serkan Burç Deşer, Berk Arapi

**Affiliations:** 1 İstanbul University-Cerrahpasa, Institute of Cardiology, İstanbul, Turkey.; 2 İstanbul University-Cerrahpasa, Medical Faculty, İstanbul, Turkey.

**Keywords:** deep vein thrombosis, seasonal variation, risk factors, trombose venosa profunda, variação sazonal, fatores de risco

## Abstract

**Background:**

Deep vein thrombosis of the lower extremities is associated with a significant burden of comorbidities.

**Objectives:**

In this study, our objective was to investigate the presence of seasonal variation in deep vein thrombosis (DVT) and assess the location of the thrombus.

**Methods:**

Out of 8177 patients admitted to two university hospitals and referred to outpatient clinics, we included a total of 611 consecutive patients (316 females, 295 males) diagnosed with acute deep vein thrombosis in this retrospective study. The mean age of the patients was 59.35±18.49 years, ranging from 1 to 96 years. Patients were categorized into four groups by age.

**Results:**

DVT was found to be more frequent in the summer (n = 190 or 31%, p = 0.003) and specifically in August (n = 65 or 10.6%, p = 0.014), while it was least frequent in the spring (n = 128 or 20.9%) and in May (n = 40 or 6.5%). However, when comparing seasons, no significant differences were observed in terms of seasonal variation (p = 0.062) or monthly variation (p = 0.143).

**Conclusions:**

Contrary to previous studies, this study demonstrated a higher occurrence of DVT during the summer, particularly in August. However, it did not reveal a clear seasonal pattern. One plausible explanation for these findings could be the adverse winter conditions and transportation challenges within the province, which may result in fewer DVT patients being able to reach hospitals for timely treatment.

## Resumo

**Contexto:**

A trombose venosa profunda (TVP) dos membros inferiores está associada a uma carga significativa de comorbidades.

**Objetivos:**

Investigar a presença de variação sazonal na TVP e identificar a localização do trombo.

**Métodos:**

Dos 8.177 pacientes internados em dois hospitais universitários e encaminhados para ambulatórios, incluímos um total de 611 pacientes consecutivos (316 mulheres, 295 homens) diagnosticados com TVP aguda neste estudo retrospectivo. A idade média dos pacientes foi de 59,35±18,49 anos, variando de 1 a 96 anos. Os pacientes foram categorizados em quatro grupos com base na idade.

**Resultados:**

A TVP foi mais frequente no verão (n = 190 ou 31%, p = 0,003), especificamente em agosto (n = 65 ou 10,6%, p = 0,014), e menos frequente na primavera (n = 128 ou 20,9%) e em maio (n = 40 ou 6,5%). No entanto, ao comparar as estações, não foram observadas diferenças significativas em termos de variação sazonal (p = 0,062) e variação mensal (p = 0,143).

**Conclusões:**

Ao contrário de estudos anteriores, este estudo demonstrou maior ocorrência de TVP no verão, principalmente em agosto. No entanto, não revelou um padrão sazonal claro. Explicações plausíveis para essas descobertas seriam as condições adversas do inverno e dificuldades de deslocamento, o que pode resultar em menos pacientes com TVP chegando aos hospitais para tratamento oportuno.

## INTRODUCTION

The incidence of many diseases is known to exhibit nonrandom distribution throughout the year. Conditions such as acute myocardial infarction, stroke, pulmonary embolism (PE), sudden cardiac death, aortic rupture, and aortic dissection have been shown to exhibit circadian, rhythmic, or seasonal patterns.^1,2^ The annual rate of first-time deep vein thrombosis (DVT) is around 100/100,000 per year and tends to increase with age.^3^ Advanced age, immobility, malignancy, history of major surgery, major trauma, prior deep vein thrombosis, and chronic heart failure are among the most common risk factors for DVT. The main mechanisms underlying DVT involve increased fibrinogen and anticardiolipin antibodies, and deficiencies in antithrombin III, protein C, and protein S, as well as activation of the coagulation system leading to hypercoagulability.^4^ The prevalence of seasonal variations in DVT and pulmonary embolism has been a topic of investigation, with some studies reporting higher rates in winter compared to summer, although no consensus has yet been reached.

Additionally, DVT has been found to peak in autumn and winter irrespective of factors such as sex, age, or event type.^5^ Moreover, acute respiratory infections are believed to increase the risk of DVT incidence and have higher occurrence in winter. Cold weather and low temperatures may contribute to changes in erythrocyte quality, increased leukocytes, granulocytes, lymphocytes, inflammation, and hypercoagulability.^6^

The objective of this study was to examine the presence of seasonal variation in deep vein thrombosis and the location of thrombi.

### Patients and methods

Out of the total of 8,177 patients who were referred to the outpatient clinic, a consecutive sample of 611 patients (316 females, 295 males) with a mean age of 59.35±18.49 years (ranging from 1 to 96 years) and diagnosed with acute deep vein thrombosis between January 2012 and November 2018 were retrospectively included in this study conducted at two tertiary centers.

The patients were categorized into different age groups: 0-40 years (group 1), 40-60 years (group 2), 60-80 years (group 3), and 80-96 years (group 4). Various factors were assessed, including gender distribution, month of diagnosis, seasonal variation in diagnosis, prevalent underlying or concomitant risk factors, and comorbid conditions such as immobilization, malignancy, pregnancy, trauma, surgery, Behçet’s disease, chronic renal failure, and the presence of coexisting pulmonary embolism. Additionally, the extent of the thrombus (iliac, femoral, popliteal, subclavian, axillary vein) and its location (upper limb or lower limb) were evaluated.

The diagnosis of deep vein thrombosis was based on the presence of signs and symptoms (swelling, pain, erythema, hyperthermia), clinical history, and laboratory, and diagnostic examinations. All patients were diagnosed using duplex Doppler ultrasonography. Unfractionated or low molecular weight heparin, new oral anticoagulant therapy (dabigatran, rivaroxaban), sodium warfarin, tight compression bandages, and stockings were administered to all patients for at least 1 year, and treatment was continued with acetylsalicylic acid, new oral anticoagulant therapy (dabigatran, rivaroxaban), or sodium warfarin throughout their lifetime. No exclusion criteria were applied in this study except including known genetic disorders and recurrent attacks. The study protocol was approved by the local ethics committee (OMÜ KAEK 2018/287) and conducted in accordance with the principles of the Helsinki Declaration.

#### Statistical analysis

The Statistical Package for the Social Sciences for Windows Version 21 (SPSS Inc., Chicago, IL, USA) was used for data comparison. The Kolmogorov-Smirnov test was employed to analyze normally distributed continuous variables. Categorical variables were presented as percentages and frequencies. Continuous variables were reported as mean ± standard deviation (SD). Independent sample *t* tests and one-way analysis of variance (ANOVA) were used to compare means for dependent groups. Continuous variables were compared using the *t* test and the Mann-Whitney U test. Categorical data were analyzed using the chi-square test or Fisher’s exact test. A p-value of <0.05 was considered statistically significant.

## RESULTS

Out of the 611 patients included in the study, 330 patients (54%) had left side DVT, 258 patients (42.2%) had right side DVT, and 23 patients (3.8%) had bilateral DVT. In terms of specific vein involvement, 384 patients (62.8%) had femoral vein thrombosis, 122 patients (20%) had iliac vein thrombosis, 68 patients (11.1%) had popliteal vein thrombosis, 22 patients (3.6%) had subclavian vein thrombosis, 12 patients (2%) had internal jugular vein thrombosis, and 3 patients (0.5%) had axillary vein thrombosis.

Among the patients, 175 (28.6%) were diagnosed with malignancy, 40 (6.5%) were immobile, 27 (4.4%) had chronic renal failure, 20 (3.3%) were pregnant, 17 (2.8%) were post-surgery, and 4 (0.7%) were diagnosed with Behcet’s Disease. No predisposing factor was identified in 327 patients (53.5%). Pulmonary embolism (PE) was present in 47 patients (7.7%), while 564 patients (92.3%) did not have accompanying PE. Table 1 provides information on the clinical, demographic, and laboratory features.

**Table 1:** The clinical, demographic, and laboratory features of patients.

	**Group I**	**Group II**	**Group III**	**Group IV**	**Total**	**P-value**
	**0-40 years**	**40-60 years**	**60-80 years**	**80-96 years**		
	**(n=103)**	**(n=172)**	**(n=252)**	**(n=84)**		
Gender (male)	49 (47.5%)	92 (53.4%)	128 (50.7%)	26 (30.9%)	295 (48.2%)	0.005
PE	7 (6.7%)	17 (9.8%)	14 (5.5%)	9 (10.7%)	47 (7.6%)	0.420
**Season of diagnosis**						
Winter	27 (26.2%)	35 (20.3%)	66 (26.1%)	22 (26.1%)	150 (24.5%)	0.516
Spring	23 (22.3%)	35 (20.3%)	52 (20.6%)	18 (21.4%)	128 (20.9%)	0.995
Summer	35 (33.9%)	60 (34.8%)	65 (25.7%)	30 (35.7%)	190 (31%)	0.127
Fall	18 (17.4%)	42 (24.4%)	69 (27.3%)	14 (16.6%)	143 (23.4%)	0.09
Diagnosis in august	11 (10.6%)	19 (11%)	26 (10.3%)	9 (10.7%)	65 (10.6%)	0.996
**Side**						
Right	56 (54.3%)	101 (58.7%)	129 (51.1%)	44 (52.3%)	330 (54%)	0.486
Left	45 (43.6%)	63 (36.6%)	114 (45.2%)	36 (42.8%)	258 (42.2%)	0.354
Bilateral	2 (1.9%)	8 (4.6%)	9 (3.5%)	4 (4.7%)	23 (3.7%)	0.665

PE = Pulmonary embolism.

When analyzing the diagnosis of DVT in relation to seasonal variation, we observed that 190 patients (31.1%) were diagnosed during summer, 150 patients (24.5%) in winter, 143 patients (23.4%) in fall, and 128 patients (20.9%) in spring. DVT was most frequently diagnosed during the summer (n = 190 or 31%, p = 0.003) and least frequently in spring (n = 128 or 20.9%). When examining the month of diagnosis, we found that DVT was most frequently diagnosed in August (n = 65 or 10.6%, p = 0.014) and least frequently diagnosed in May (n = 40 or 6.5%). When comparing the seasonal variation specifically in females, we discovered a statistically significant correlation (p = 0.013). However, no statistical significance was observed in males (p = 0.086) or across the patient group as a whole (p = 0.062)

Furthermore, when comparing patients based on seasonal variation, no statistically significant differences were found in terms of platelet count (p = 0.518), mean platelet volume (MPV) (p = 0.324), presence of pulmonary embolism (PE) (p = 0.070), location of DVT (p = 0.769), side of DVT (p = 0.483), or primary diagnosis (p = 0.337). When classifying patients into four age groups, we identified that 103 patients belonged to Group 1 (0-40 years), 172 patients to Group 2 (40-60 years), 252 patients to Group 3 (60-80 years), and 84 patients to Group 4 (80-96 years). When comparing these four age groups, no statistically significant differences were observed in terms of seasonal variation (p = 0.294), monthly variation (p = 0.309), DVT location (p = 0.301), side (p = 0.607), presence of PE (p = 0.420), platelet count (p = 0.282), or mean platelet volume (MPV) (p = 0.284). However, we did find significant differences in terms of primary diagnosis (p = 0.001, malignancy), type of malignancy (p = 0.001), and gender distribution (p = 0.005). Specifically, leukemia was more frequently observed in patients aged 60-80 years across all age groups. Table 2 shows the comparison of the groups based on age.

**Table 2:** Comparison of groups according to age.

	**Group I**	**Group II**	**Group III**	**Group IV**	**Total**	**P-value**
	**0-40 years**	**40-60 years**	**60-80 years**	**80-96 years**		
	**(n=103)**	**(n=172)**	**(n=252)**	**(n=84)**		
**Location**						
Iliac	22 (21.3%)	27 (15.6%)	58 (23%)	15 (17.8%)	122 (19.9%)	0.285
Femoral	70 (67.9%)	107 (62.2%)	161 (63.8%)	55 (65.4%)	384 (62.8%)	0.801
Popliteal	14 (13.5%)	23 (13.3%)	22 (8.7%)	9 (10.7%)	68 (11.1%)	0.393
Subclavian	1 (0.9%)	8 (4.6%)	9 (3.5%)	4 (4.7%)	22 (3.6%)	0.403
Axillary	1 (0.9%)	2 (1.1%)	0	0	3 (0.4%)	0.145
Jugular	4 (3.8%)	5 (2.9%)	2 (0.7%)	1 (1.1%)	12 (1.9%)	0.185
**Total**	103	172	252	84	611	
Malignancy	10 (9.7%)	56 (32.5%)	89 (35.3%)	20 (23.8%)	175 (28.6%)	0.001
Pregnancy	17 (16.5%)	2 (1.1%)	1 (0.39%)	0	20 (3.2%)	0.254
Post surgery	4 (3.8%)	7 (4%)	4 (1.5%)	2 (2.3%)	17 (2.7%)	0.407
Immobility	5 (4.8%)	9 (5.2%)	15 (5.9%)	11 (13%)	40 (6.5%)	0.071
Behçet’s disease	3 (2.9%)	1 (0.5%)	0	0	4 (0.6%)	0.016
Chronic renal failure	3 (2.9%)	6 (3.4%)	10 (3.9%)	8 (9.5%)	27 (4.4%)	0.101
**Total**	103	172	252	84	611	
Platelet count	261.80±15.8	246.3±10.5	232.05±9.3	228.6±12.7	0.282	
MPV	8.93±1.52	8.85±1.62	8.99±1.58	9.21±1.25	0.284	

MPV = mean platelet volume.

When comparing these groups specifically in terms of male gender, no statistical differences were found in relation to seasonal variation (p = 0.91), monthly variation (p = 0.260), side (p = 0.846), primary diagnosis (p = 0.161), presence of pulmonary embolism (PE) (p = 0.819), or platelet count (p = 0.051). However, we did observe statistical differences regarding mean platelet volume (MPV) count (p = 0.017), DVT location (p = 0.008), and type of malignancy (p = 0.001). Specifically, lung and prostate cancers were the most common etiologies among men aged 60 to 80 years. The femoral location was the most frequently observed, and MPV levels were higher.

When comparing the age groups specifically in terms of the female sex, no statistically significant differences were observed regarding seasonal variation (p = 0.484), monthly variation (p = 0.082), DVT location (p = 0.578), side (p = 0.352), presence of pulmonary embolism (PE) (p = 0.418), platelet count (p = 0.684), or mean platelet volume (MPV) count (p = 0.225). However, statistical differences were found in terms of primary diagnosis (p = 0.001), favoring malignancy, and for the type of malignancy (p = 0.019).

## DISCUSSION

The main findings of this study were that no clear seasonal pattern was observed, although DVT appeared to be more common in the summer (especially August) and less common in the spring (especially May). Additionally, it was found that all patients with lower limb DVT had above-knee thrombosis and the majority of patients diagnosed with deep vein thrombosis were between the ages of 60 and 80. Leukemia was identified as the leading cause among patients, particularly in the age range of 60 to 80. Additionally, lung and prostate cancers were the most prevalent etiologies among males in the same age group, and they were associated with higher MPV levels. The femoral region was the most frequently affected location in cases of deep vein thrombosis (DVT).

The understanding of the seasonal pattern of DVT in relation to hospital admissions has been a subject of debate in some studies. Several mechanisms were proposed as contributing to the seasonal pattern of DVT. Most of the studies conducted on this topic were retrospective and carried out at single centers. The role of seasonal variation was found to be around 2% and some authors have found correlations between season and DVT and chronobiological patterns (circadian, weekly, and seasonal).^7^ Known predisposing factors for DVT include advanced age (>40 years), previous history of DVT or PE, malignancy, immobilization, major surgery, congestive heart failure, chronic venous insufficiency, oral contraceptive use, stroke, multiple traumas, delivery or pregnancy, and myocardial infarction.^4^ While the incidence of pulmonary embolism has been reported to decrease with advanced age, the incidence of DVT has remained constant in males. However, there has been a rise in the incidence of DVT among elderly females.^7^ Several authors have shown a simultaneous seasonal occurrence of DVT and cardiovascular disease. Vitamin D has been found to be associated with fibrinolytic and procoagulant activity.^8^ The relative hypercoagulability observed during colder weather further increases the risk of DVT, as seasonal variation elevates fibrinogen levels, leading to hypercoagulability.^4,6,9-11^

It is suggested that cold weather may induce vasoconstriction, resulting in reduced blood flow in the veins of the lower limbs due to decreased physical activity, while respiratory tract infections during winter may also lead to fluctuations in coagulation, contributing to the seasonal variation. Additionally, it has been hypothesized that temperature fluctuations and chilling may influence cardiovascular mortality.^6^ Manfredini et al.^12^ reported that the presence of DVT was independent of underlying coagulation disorders. They found higher values of fibrinogen, FVIIc plasma, and protein C and protein S insufficiency in patients diagnosed with DVT during the winter. Other authors have proposed that reduced physical activity and a sedentary lifestyle during colder months may contribute to the development of DVT.^13^ In addition, a significant seasonal variation was reported in a study by Zhao et al.,^14^ with the highest occurrence observed in December among a population consisting of both males and females. Additionally, Masotti et al.^15^ reported that during the winter, C-reactive protein, D-dimer, and platelet levels were found to be higher, leading to a relative hypercoagulable state. Jang et al.^13^ conducted a study with 1495 patients diagnosed with DVT and observed a significant peak during the winter, particularly in January.

However, studies conducted by Dentali et al.,^3^ Fink et al.,^7^ Lee and Levine,^16^ and Damnjanović et al.^17^ reported no correlation between seasonal, monthly, or climatic factors and deep vein thrombosis. Furthermore, some authors have reported a rhythmic pattern with DVT being more frequent in autumn and less frequent in spring. They also noted that DVT was most commonly observed in patients with a history of previous DVT, in immobile patients, and in males over 40 years old.^13^

In contrast to previous studies, our findings indicated that DVT was more frequent in the summer and less frequent in the spring. We hypothesized that patients in our region may be less likely to seek hospital admission during cold weather due to geographical conditions. Despite this assumption, we did not find any evidence of seasonal variation in our study.

However, Lan Chang et al.^18^ reported that temperature changes of 5°C were not found to be significantly related to the incidence of DVT. The role of seasonal variation in specific patient populations during high-risk periods is considered important for appropriate and effective DVT prophylaxis.

Damnjanović et al.^19^ reported a significant relationship between DVT and atmospheric pressure, temperature, and humidity, suggesting that changes in viscosity and coagulation may play a role. However, the exact mechanism behind the seasonal or monthly variations in the existence of DVT is still not fully understood. One distinguishing aspect of our study compared to others is the evaluation of primary etiology, particularly focusing on malignancy. We found that leukemia was the most common etiology among patients with DVT, especially in those aged between 40 and 60 years old. Additionally, we observed that femoral region DVT and lung and prostate cancers were most frequently seen in males aged 60-80 years old.

In a study conducted by Fink et al.,^7^ which included 905 patients, below-knee thrombosis was more frequent in winter, while above-knee thrombosis was more frequent in the warmer months (April-September). Our results were partially consistent with this study in that we found DVT to be more frequent in summer. However, none of our patients with lower limb DVT had isolated below-knee thrombosis, which may have influenced our results.

Our study has several limitations. Firstly, it has a retrospective design, which may introduce potential biases. Secondly, the sample size of our study may appear limited compared to other studies, which could affect the generalizability of our findings. Thirdly, larger studies would be necessary to validate and replicate our results. Fourthly, the power of our outcomes might have been reduced as it was a two-center study. Fifthly, we investigated a selective patient sample, which may limit the generalizability of our findings.

In conclusion, our study did not find any significant variation in the location of thrombus based on seasonal or monthly factors. However, we observed a higher frequency of DVT diagnoses during the summer, although this finding did not reach statistical significance. Additionally, we found that DVT was most frequently diagnosed in patients aged between 60 and 80 years old, particularly in the femoral region. One possible explanation for these findings could be the challenging winter conditions and transportation difficulties that may affect healthcare seeking behavior during colder months.
